# IOA-244, a novel p110δ PI3K inhibitor, blocks breast tumour progression on either mono- or combined-therapy

**DOI:** 10.1038/s41420-026-03073-3

**Published:** 2026-03-27

**Authors:** Evangelia Goulielmaki, Anna Tsapara, Lydia Xenou, Zoe Johnson, Karolina Niewola-Staszkowska, Maria Tzardi, Eelco de Bree, Evangelia A. Papakonstanti

**Affiliations:** 1 Department of Biochemistry, School of Medicine, University of Crete, Heraklion, Greece; 2 iOnctura SA, Campus Biotech Innovation Park, Genève, Switzerland; 3 Department of Pathology, University Hospital, School of Medicine, University of Crete, Heraklion, Greece; 4 Department of Surgical Oncology, University Hospital, School of Medicine, University of Crete, Heraklion, Greece

**Keywords:** Breast cancer, Drug development

## Abstract

The clinical approval of p110δ PI3K inhibitors raised hopes in treating aggressive tumours expressing high levels of non-mutated p110δ, however, the severe adverse effects that those inhibitors caused became a barrier to their clinical application. IOA-244 is the first-in-class, highly selective and non-ATP competitive p110δ PI3K inhibitor showing high selectivity and low toxicity in several preclinical models. Here we show that IOA-244, as a single agent treatment, blocks the progression of early phase breast tumours by attacking the survival of cancer cells and the polarisation of TAMs to a pro-tumourigenic phenotype leading to suppression of TAMs-expressed ATX. In established tumours, IOA-244 alone was insufficient to control the high levels of both M2-like macrophages and ATX, and while it reduced tumour progression, it did not completely block it. Full tumour control, however, was achieved when IOA-244 used in a combinatorial regimen with the PF-8380 ATX inhibitor. In agreement with the mouse model, the amount of CD163+/CD204+macrophages and ATX were much higher in grade III human breast carcinomas compared to grade I. Our work provides the first in vivo preclinical evidence showing that IOA-244 is a potential highly effective drug for breast cancer treatment and depending on the phase of the tumour can be used either as a single agent or as a combinatorial regimen.

## Introduction

The phosphoinositide 3-kinase (PI3K) signalling pathway regulates essential cellular functions and is strongly associated with cancer, with class I PI3Ks extensively investigated in human malignancies [1–3]. The class IA subgroup of PI3Ks is activated by growth factor receptor tyrosine kinases (RTKs) and consists of the 110 kDa catalytic subunits p110α, p110β and p110δ, each forming heterodimers with the regulatory subunit p85 [3, 4]. Given the involvement of class IA PI3Ks in cancer initiation and progression [5] a broad range of PI3K inhibitors has been clinically developed for the treatment of various malignancies [6].

The gene encoding p110δ PI3K is rarely mutated in cancers [7–9]. Because p110δ is predominantly expressed in leucocytes, this isoform has been extensively investigated in the contexts of immunity, inflammation, and haematologic malignancies [10–16]. More recently, a promising role for p110δ PI3K has emerged in solid tumours that express high levels of the non-mutated protein. Strong evidence indicates that p110δ contributes both to cancer cell–intrinsic oncogenic functions and to stromal, tumour-supporting mechanisms [3, 17–19]. Mouse xenograft studies have shown that the sensitivity of solid tumours to p110δ-selective inhibitors is driven by cancer cell–intrinsic p110δ activity, which functions as an oncogenic driver [3, 17, 18]. In addition, numerous studies have demonstrated that inhibition of p110δ exerts potent immunomodulatory effects by suppressing regulatory T cells (Tregs) and myeloid-derived suppressor cells (MDSCs), thereby enhancing effector T cell (Teff) activity [20–23]. Inactivation of p110δ also reduces tumour-associated macrophages (TAMs), contributing to the suppression of solid tumour growth [17]. Other findings further show that p110δ PI3K inhibition prevents the death of natural killer (NK) cells by limiting the ability of TAMs to generate reactive oxygen species [24].

The growing recognition of the pivotal role of p110δ PI3K in cancer has driven the development of multiple p110δ inhibitors that have undergone clinical evaluation, demonstrating notable anti-tumour efficacy. Idelalisib (Zydelig™; CAL-101; Gilead Sciences) was the first-in-class small-molecule inhibitor with high selectivity for p110δ and was approved for the treatment of chronic lymphocytic leukaemia (CLL), relapsed follicular B-cell non-Hodgkin lymphoma (FL), and relapsed small lymphocytic lymphoma (SLL) [25, 26]. Duvelisib (Copiktra™; IPI-145; Verastem), a selective dual inhibitor of p110δ and p110γ, has shown significant efficacy as monotherapy for CLL/SLL patients [27] and as treatment of adult patients with relapsed or refractory CLL, SLL and FL [28]. Umbralisib (UKONIQ™; TGR-1202; TG Therapeutics) is also a dual inhibitor which is highly selective for p110δ inhibiting also casein kinase 1epsilon (CK1ε) and was used for the treatment of adult patients with relapsed or refractory marginal zone lymphoma (MZL) [29, 30]. Although several p110δ PI3K inhibitors have shown remarkable anti tumour activity and received approval from the Food and Drug Administration (FDA), the immunomodulation caused by those inhibitors resulted in severe adverse effects that became a barrier to their clinical application. Therefore, the FDA published a warning opinion on the toxicity profile of these inhibitors that led to voluntarily withdrawal of idelalisib for treatment of FL and SLL [31], duvelisib for FL and umbralisib for both MZL and FL due to increased risk of death for patients [6, 32].

All p110δ PI3K inhibitors have been designed to bind to the ATP pocket except for the IOA-244, the first non-ATP competitive p110δ inhibitor, developed by iOnctura, and efficacious in various preclinical cancer models [33]. Furthermore, IOA-244 was found to inhibit the proliferation of T_reg_ cells with limited effects on CD4^+^ T cells and no effects on CD8^+^ T cells in colorectal and lung cancer models [34] whereas in combination therapy with either anti-PD1 or anti-PD-L1 significantly inhibited tumour growth in pancreatic and lymphoma syngeneic mouse models [35]. It is of note that the use of IOA-244 was followed by remodelling of the tumour microenvironment in those models, showing high selectivity and low toxicity. IOA-244 is in clinical Phase I/II studies in patients with solid tumours and haematological malignancies [35].

Breast cancer shows a wide variability in its development, progression and resistance to chemotherapy or radiotherapy, raising the need for additional targeted therapeutic approaches. The ATX-LPA axis has been strongly correlated with induced inflammation, tumour growth, metastasis and chemo-resistance [36–39]. In fact, autotaxin (ATX) is a secreted enzyme which converts extracellular lysophosphatidylcholine (LPC) into lysophosphatidate (LPA) [40, 41] which then activates at least six G protein coupled receptors to increase cell division, survival and migration, by stimulating a number of signalling pathways including that of PI3K [42–45], of both tumour cells as well as cells in the tumour microenvironment. The levels of ATX are elevated in many tumours, however, breast cancer cells are considered poor producers of ATX [46, 47] compared to cells of the tumour surrounding stroma [40, 46, 48]. The ATX-LPA signalling though is known to play an important role in breast cancer because the ATX produced by the tumour surrounding tissues stimulates breast cancer cells to secrete cytokines which in turn stimulate the adjacent tissues to produce more ATX [37–39, 49, 50]. Thus, the increased positive feedback loop of ATX increases tumour LPA concentrations, sustaining a cycle that fuels tumour growth and metastasis.

In the current study, we have examined the potential effectiveness of IOA-244 starting treatment at two different time-points following cell inoculation: in early-phase tumours and in established tumours. We show that IOA-244 given at the early phase reduces the survival of breast cancer cells and decreases the abundance of M2-like macrophages in tumour sites, leading to diminished expression of TAMs-expressed ATX in early developed breast tumours and consequently blocking tumour progression. In established breast cancer tumours, IOA-244 was found to stem the numbers of M2-like macrophages and the overexpression of ATX leading to significant but not complete reduction of the tumour burden. However, a combination of IOA-244 and an inhibitor of ATX was found to be strikingly effective in entirely abrogating tumour burden progression, arguing in favour of such a dual-pronged approach in treating challenging solid tumours.

## Results

### Validation of the efficacy of IOA-244 in modulating proliferation and apoptosis of breast cancer cells in vitro

Our previously reported findings have shown that the p110δ is the predominant PI3K isoform, among p110α and p110β, in MDA-MB-231 breast cancer cells and in primary human breast carcinomas and that p110δ selective inhibition has a dominant effect on Akt phosphorylation and tumour cell proliferation over that of p110α- or p110β- selective inhibitors [18]. In line with this published data, inhibition of p110δ by IOA-244 also dose-dependently decreased the phosphorylation of Akt (Supplementary Fig. 1A) comparable to Idelalisib (Supplementary Fig. 1B), confirming the efficacy of IOA-244 as a p110δ inhibitor on breast cancer cells in vitro. We further evaluated the impact of IOA-244 on proliferation and apoptosis and on potential toxicity of this inhibitor on MDA-MB-231 cells. The expression of the proliferation marker Ki-67 was decreased over increasing concentrations of IOA-244 with the first significant decrease to be observed at 5 μM (Supplementary Fig. 1C) whereas the apoptosis marker cleaved caspase-3 (Supplementary Fig. 1D) and the activity of caspase 3/7 (Supplementary Fig. 1E) were strongly increased even at 2 μM of IOA-244. Moreover, the release of LDH, a marker for cytotoxicity and necrosis [51], in the culture medium of MDA-MB-231 cells remained constantly low upon treatment of cells with IOA-244 even at higher concentrations (Supplementary Fig. 1F) indicating low toxicity of this p110δ inhibitor.

The above results suggested that IOA-244 might also have a strong effect on breast cancer progression in vivo.

### Oral administration of the IOA-244 p110δ−selective inhibitor blocks the growth of early phase breast tumours and regresses the tumour burden progression in established tumours, affecting the survival of both, TAMs and cancer cells and cancer cells metastasis

We then evaluated the impact of IOA-244 on the progression of breast cancer tumours in vivo. To do this, we inoculated the MDA-MB-231 triple-negative breast cancer cell line into Balb/c nude mice to generate mammary tumours. Breast cancer staging ranges from stage 0 through IV (4) with the stage to depend on the size of the tumour, along with several other factors and traditionally increased tumour size at diagnosis is associated with an increased risk of distant metastases and mortality [52]. Stage 0 corresponds to in situ carcinomas that have not spread from the location where they first formed and have no potential for metastasis whereas stage IV corresponds to metastatic cancers that have spread outside the first location to other parts of the body. The tumour stages (hereinafter referred to as ‘phases’) in mice models of triple-negative breast cancer cell lines have been characterised as early phase for tumour volume <100 mm^3^, intermediate phase for tumour volume 100-300 mm^3^, advanced phase for tumour volume 300–500 mm^3^, and end phase for tumour volume >500 mm^3^ [53] which is analogous to the established staging of human breast cancers [54–56]. Therefore, we have considered (and refer to them) as ‘early phase’ the tumours that developed for 12 days after cancer cells inoculation (tumour volume <100 mm^3^ and as ‘intermediate-established phase’ the tumours that grew for 20 days after cancer cells inoculation and reached an intermediate phase with tumour volume between 100 and 300 mm^3^.

**Fig. 1:**
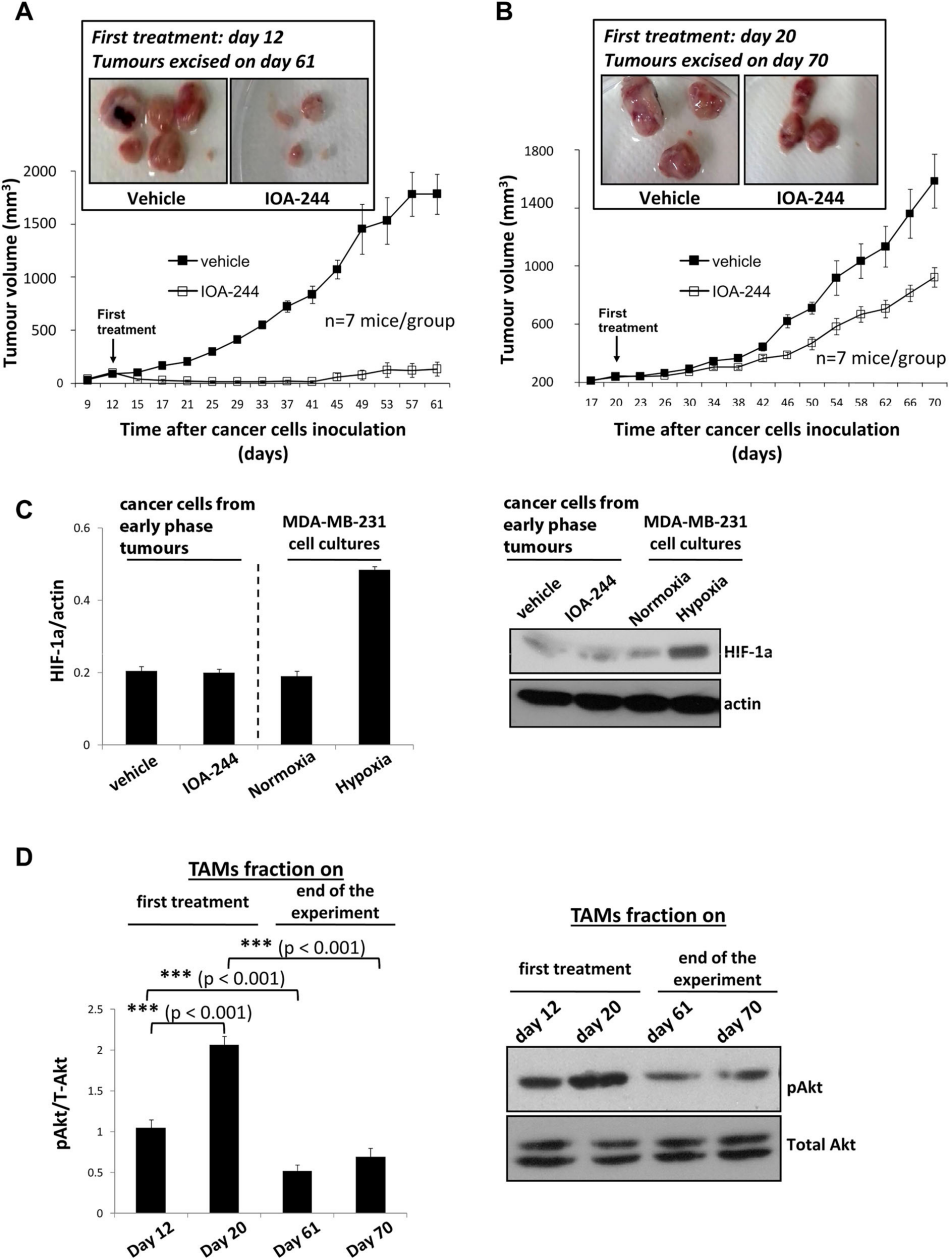
Impact of IOA-244 p110δ-selective inhibitor on tumour growth, necrosis of tumour-isolated cancer cells and survival of TAMs of early developed and established breast tumours. **A** BALB/c nude mice were inoculated with MDA-MB-231 breast cancer cells in the breast fat pad on day 0 and treated twice daily *per os* with vehicle or IOA-244 (30 mg/kg) from day +12. The tumours were measured by digital callipers and expressed as tumour volume. *n* = 7 mice/group. Inset: tumours excised from mice that were treated with IOA-244 (30 mg/kg) from day +12 and mice treated with vehicle. **B** BALB/c nude mice were inoculated with MDA-MB-231 breast cancer cells in the breast fat pad on day 0 and treated twice daily *per os* with vehicle or IOA-244 (30 mg/kg) from day +20. The tumours were measured by digital callipers and expressed as tumour volume. *n* = 7 mice/group. Inset: tumours excised from mice that were treated with IOA-244 (30 mg/kg) from day +20 and mice treated with vehicle. **C** At the end day (day 61) of the experiment described under “A”, cancer cells were isolated from tumours excised from mice that were treated with IOA-244 or vehicle and necrosis was determined by assessing HIF-1a by Western blotting of total cell lysates. As a positive control experiment, HIF-1a expression was assessed in MDA-MB-231 cells under normoxia or hypoxia. **D** The TAMs fraction was isolated from tumours excised the first treatment day (day +12 or day +20) and the last day (day +61 or day +70) of the experiments described under ‘A’ and ‘B’ and the phosphorylated Akt was determined by Western blotting of total cell lysates. All graphs represent means ± s.e.m. Statistically significant differences are indicated by *** (*P* < 0.001), as determined by the Mann-Whitney test.

Per os twice daily administration of IOA-244 (from day +12, i.e. when mouse tumours were at an early phase)) led to an almost complete block of tumour growth (Fig. 1A) and in a great reduction of the mass in tumours harvested at day +61 (Fig. 1A, inset). We then explored whether IOA-244 has the same impact on intermediate-phase breast tumours. To explore this possibility, we tested the impact of similar oral administration of IOA-244 starting at day +20 on tumour growth in MDA-MB-231-bearing Balb/c nude mice. Under these conditions, in contrast, IOA-244 reduced but did not entirely block tumour burden (Fig. 1B). The modest effect on tumour growth rate was also reflected in a modest decrease in tumour mass harvested from mice at day +70 (Fig. 1B, inset).

To examine whether the strong effect of IOA-244 on early phase tumours is a result of IOA-244-induced necrosis, we evaluated the expression of HIF-1α, given that necrosis is reflected by an intratumoural hypoxic environment [57, 58], in cancer cells isolated from excised tumours (Fig. 1C). The results showed that the expression of HIF-1α was not affected by IOA-244 treatment (Fig. 1C) indicating that the blockade of tumour growth was not because of a necrotic cell death which is in line with the in vitro experiment showing that the LDH levels in culture medium of MDA-MB-231 cells kept low upon IOA-244 treatment (Supplementary Fig. 1F). Culturing MDA-MB-231 cells under normoxic or hypoxic conditions, as a control experiment, revealed that the expression of HIF-1α was significantly promoted under hypoxic conditions (Fig. 1C).

To evaluate a potential role of tumour-associated macrophages (TAMs) in tumour growth regression under each treatment condition, we determined the effect of IOA-244 on Akt phosphorylation in TAMs fraction isolated from excised either early or intermediate phase tumours on the first treatment day (day +12 or day +20) and the last day of the experiment (day +61 or day +70). Interestingly, we found that the phosphorylation of Akt the first treatment day was increased in TAMs fraction of intermediate compared to that in early phase tumours, however, the levels of phosphorylated Akt were similarly decreased in TAMs fraction of tumours that were excised from mice at the end of both experiments compared with the phosphorylated Akt in TAMs fraction of tumours excised the respective first treatment day (Fig. 1D). These results suggested that a reduction in survival of TAMs contributes to suppression of tumour growth by IOA-244, however, additional functions or factors seem to affect the different outcomes of the two treatment conditions (i.e. treatment started when tumours were at an early or established phase).

**Fig. 2:**
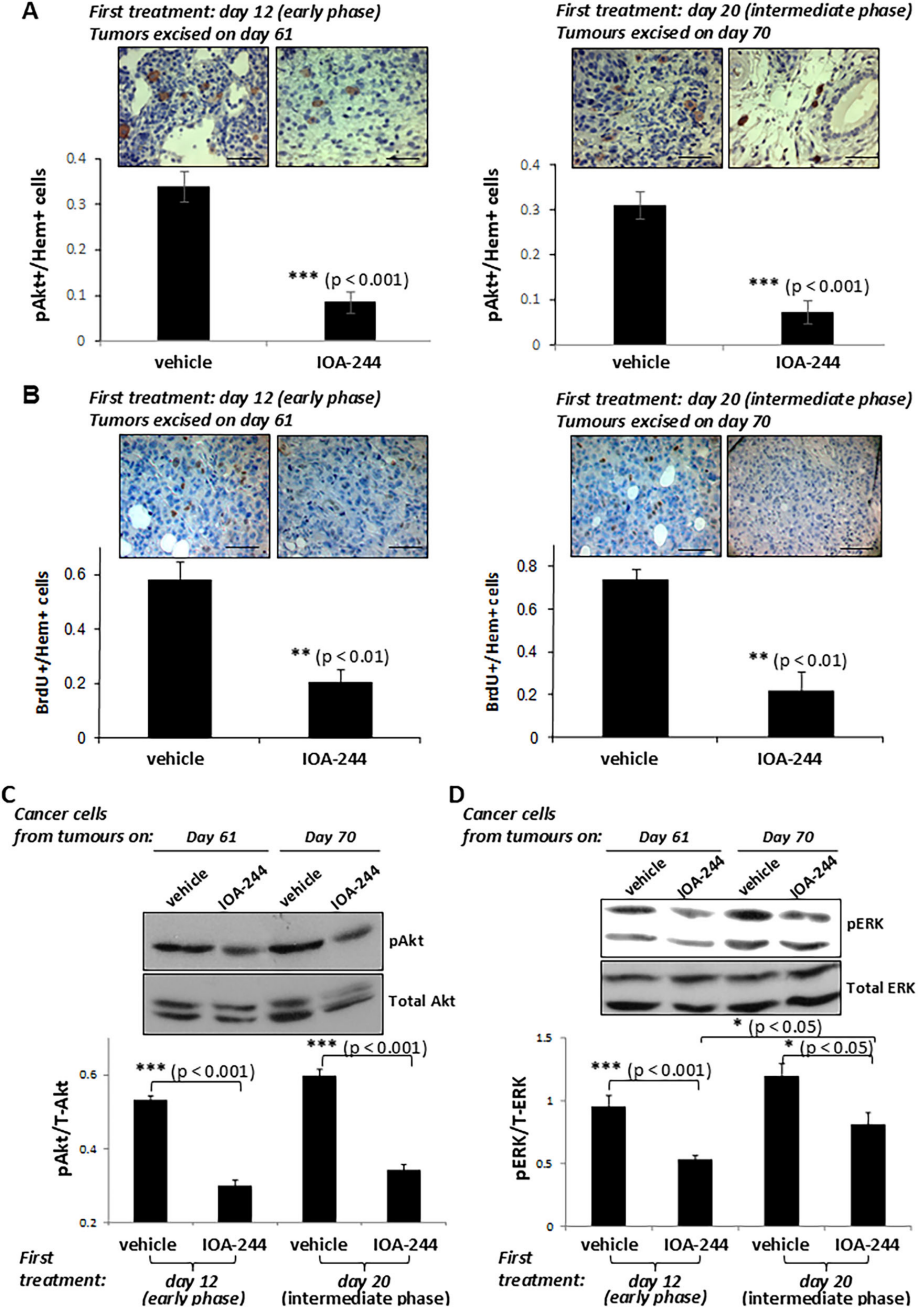
The IOA-244 p110δ-selective inhibitor strongly affects the survival and proliferation of breast tumour cells. **A** MDA-MB-231 primary tumours harvested from BALB/c nude mice that were treated with IOA-244 or vehicle from day +12 to day +61 (left panel) or from day +20 to day +70 (right panel) were subjected to immunohistochemical analysis using anti p-Akt antibody (brown) and Hematoxylin (blue) followed by comparison of p-Akt positive (pAkt+/Hem+) cells in tumours of IOA-244 -treated and vehicle-treated mice. Scale bar= 50 μm. *n* = 7 mice/group. **B** Cell proliferation in tumours excised from mice that were treated with IOA-244 or vehicle from day +12 to day +61 (left panel) or from day +20 to day +70 (right panel) was determined by BrdU incorporation (brown spots) followed by comparison of BrdU-positive (BrdU + /Hem + ) cells in tumours of IOA-244-treated and vehicle-treated mice. Scale bar = 50 μm. *n* = 7 mice/group. **C** Cancer cells were isolated from tumours excised the last day (day +61 or day +70) of the experiments in mice that were treated with IOA-244 or vehicle either from day +12 to day +61 or from day +20 to day +70 and the phosphorylated Akt was determined by Western blotting of total cell lysates. **D** Cancer cells were isolated from tumours excised the last day (day +61 or day +70) of the experiments in mice that were treated with IOA-244 or vehicle either from day +12 to day +61 or from day +20 to day +70 and the phosphorylated ERK was determined by Western blotting of total cell lysates. All graphs represent means ± s.e.m. Statistically significant differences are indicated by * (*P* < 0.05), ** (*P* < 0.01) or *** (*P* < 0.001), as determined by the Mann-Whitney test.

We then sought to determine the direct effect of IOA-244 on tumour cells in vivo by investigating first whether those treatments affect the survival and proliferation rate of tumour cells. The phosphorylation of Akt in excised tumours at the end of the experiments was effectively reduced by IOA-244 under both treatment conditions compared with that from tumours excised from untreated mice (Fig. 2A), correlating with the ability of IOA-244 to reduce cell survival. The BrdU-positive cells (Fig. 2B) were also significantly reduced in specimens excised from mice that started receiving IOA-244 when bearing either early or intermediate phase tumours compared with mice receiving the vehicle only, indicating that the proliferative rate of tumours was prevented similarly under both treatment schedules. To confirm more accurately the effect of IOA-244 on cancer cells, we isolated the cancer cells from excised tumours and determined the phosphorylated Akt as well as the phosphorylated ERK1/2, which is critical in transmitting signals from cell membrane to the nucleus [59] and both pathways, that of Akt and ERK1/2, have a vital role in promoting cell apoptosis [60]. In agreement with the results obtained by the immunohistochemistry experiments (Fig. 2A), the levels of phosphorylated Akt were similarly decreased in cancer cell fractions of tumours that were excised from mice at the end (day +61 and day +70) of both experimental conditions compared with that from the respective control mice that were treated with the vehicle (Fig. 2C). In contrast, the phosphorylation of ERK1/2 was decreased less in cancer cells from excised tumours from mice started receiving IOA-244 when bearing intermediate phase tumours than that in those bearing early phase tumours compared with the respective untreated mice (Fig. 2D). Furthermore, the phosphorylation of ERK1/2 at the end of the experiment (day +70) in intermediate phase tumours was increased compared with that at the end of the experiment (day +61) in early phase tumours (Fig. 2D).

These results indicate that a signal that is possibly transmitted from the cell membrane of cancer cells through ERK might be stronger when the treatment with IOA-244 starts in mice with established-intermediate tumours compared with that in mice with early phase tumours making the IOA-244 inhibitor less effective.

**Fig. 3:**
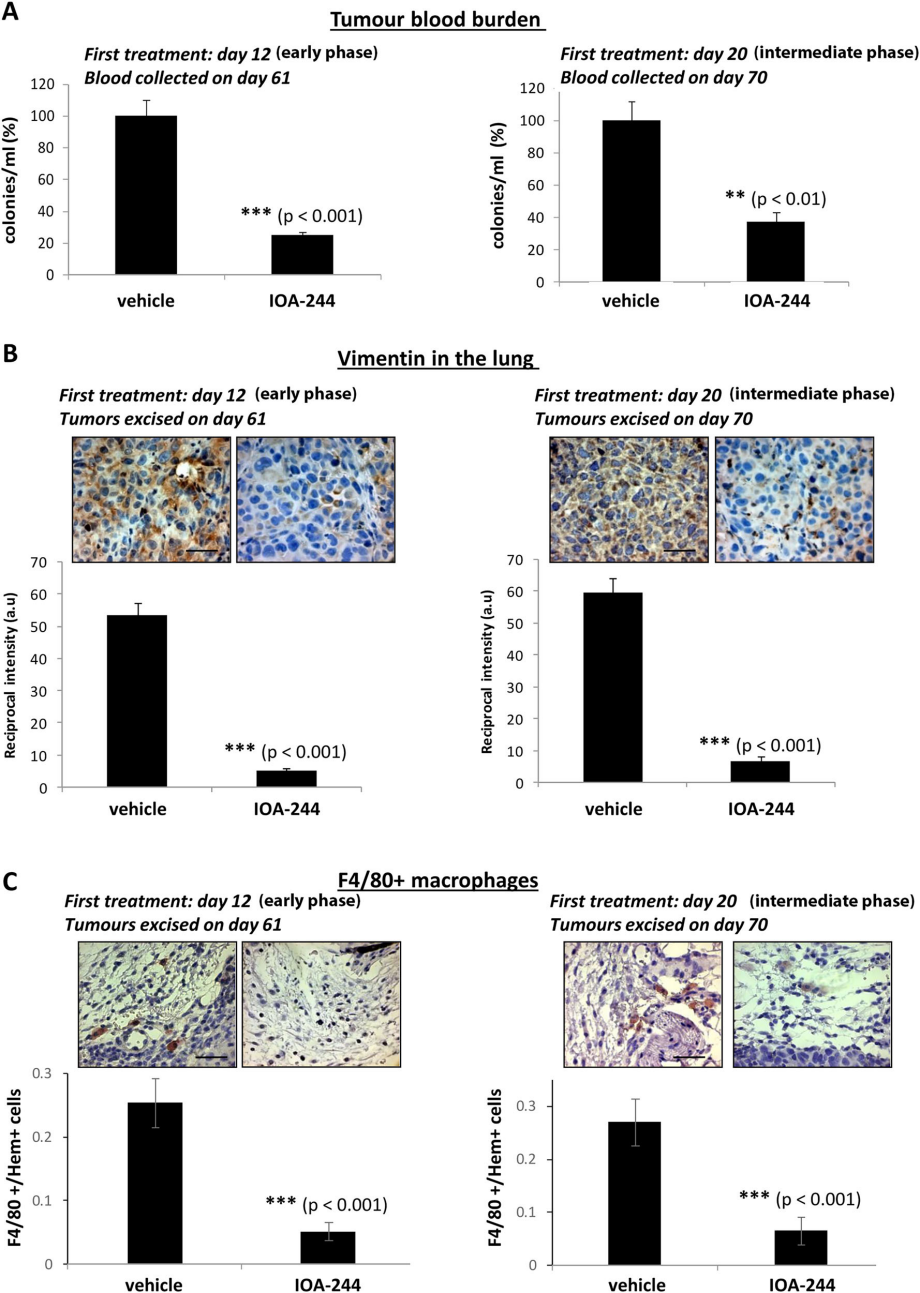
Impact of IOA-244 on metastasis of breast tumour cells and on the recruitment of macrophages to tumour sites. **A** Intravasation efficiency of cancer cells as determined by tumour cells blood burden at the end point of the experiment in BALB/c nude mice which were treated with IOA-244 or vehicle from day +12 (left panel) or from day +20 (right panel). *n* = 7 mice/group. **B** Invasion of cancer cells as determined by immunohistological staining of vimentin (brown) in the lungs of BALB/c nude mice which were treated with IOA-244 or vehicle from day +12 (left panel) or from day +20 (right panel). *n* = 7 mice/group. Scale bar = 50 μm. **C** MDA-MB-231 primary tumours harvested from BALB/c nude mice that were treated with IOA-244or vehicle from day +12 (left panel) or from day +20 (right panel) were subjected to immunohistochemical staining of macrophage-specific antigen F4/80 (brown) and Hematoxylin (blue) followed by comparison of F4/80 positive cells in tumours of IOA-244-treated and vehicle-treated mice. Scale bar = 50 μm. All graphs represent means±s.e.m. Statistically significant differences are indicated by ** (*P* < 0.01) or *** (*P* < 0.001), as determined by the Mann-Whitney test.

We then assessed the impact of the two different treatment schedules with IOA-244 on cancer cell metastasis by determining the tumour cell blood burden and the expression of vimentin in the lungs. The tumour cells number in the blood collected from the right atrium of the heart before filtration by the lungs, is a direct evaluation of intravasation and therefore of efficiency of metastatic dissemination [61], whereas elevated expression of vimentin correlates with lung invasion of cancer cells [62, 63]. IOA-244 significantly diminished the tumour cell blood burden (Fig. 3A) as well as the expression of vimentin in the lungs (Fig. 3B) indicating high effectiveness of IOA-244 in preventing the spontaneous intravasation and dissemination of breast cancer cells as well as to decrease their spread in other organs. It is of note that similar reduction in the tumour blood burden (Fig. 3A) and the expression of vimentin in the lungs (Fig. 3B) was achieved whether the inhibitor was administered *per os* in early phase (from day +12) (Fig. 3A, B, left panels) or in intermediate phase tumours (from day +20) (Fig. 3A, B, right panels). In addition, immunostaining of tumour samples with the macrophage-specific antigen F4/80 showed that the abundance of macrophages into tumour sites at the end of the experiments were reduced almost equally under both conditions, either when treatment with IOA-244 started on day +12 (early phase tumours) or day +20 (intermediate phase tumours) compared with that in mice treated with the vehicle (Fig. 3C) which is in line with the equal impact of IOA-244 on the phosphorylation of Akt in TAMs fraction (Fig. 1D) and on metastasis (Fig. 3A, B) under both conditions.

Taken together, the above results demonstrate that the IOA-244 p110δ-selective inhibitor directly affects the survival of TAMs as well as the survival and proliferation of breast cancer cells making up the tumour preventing their invasive activity and metastasis, independently of the onset of administration. However, the fact that the phosphorylation of ERK was less affected by IOA-244 in cancer cells from intermediate phase tumours than that from early phase tumours makes it likely that additional cell functions and/or molecular factor(s) in breast tumour microenvironment might counterbalance the effect of IOA-244 on tumour growth of established-intermediate tumours.

### The efficacy of IOA-244 treatment correlates with its effect on the abundance of M2-like macrophages and the TAM-expressed ATX

It is known that ATX induces tumour growth and metastasis, reduces the efficacy of chemotherapy and radiotherapy [39, 40] and these effects are independent of breast cancer type since ATX is in major part not derived from breast cancer cells [46, 47]. In breast cancer, however, macrophages have been found to play a seminal role in tumour progression [64, 65], to influence the response to cancer therapies [66, 67] and furthermore the CD163+CD206+ TAMs have been considered as the primary source of ATX [46, 68]. We showed above that IOA-244 exerts a valuable effect on the survival of TAMs which is in line with our previously reported data showing that the first small molecule inhibitor with selectivity for p110δ [69], blocks breast tumour development and metastasis in mice models by targeting cancer cells and macrophages [17], however, the impact of p110δ inactivation on the polarisation of macrophages in breast cancer remains unexplored.

**Fig. 4:**
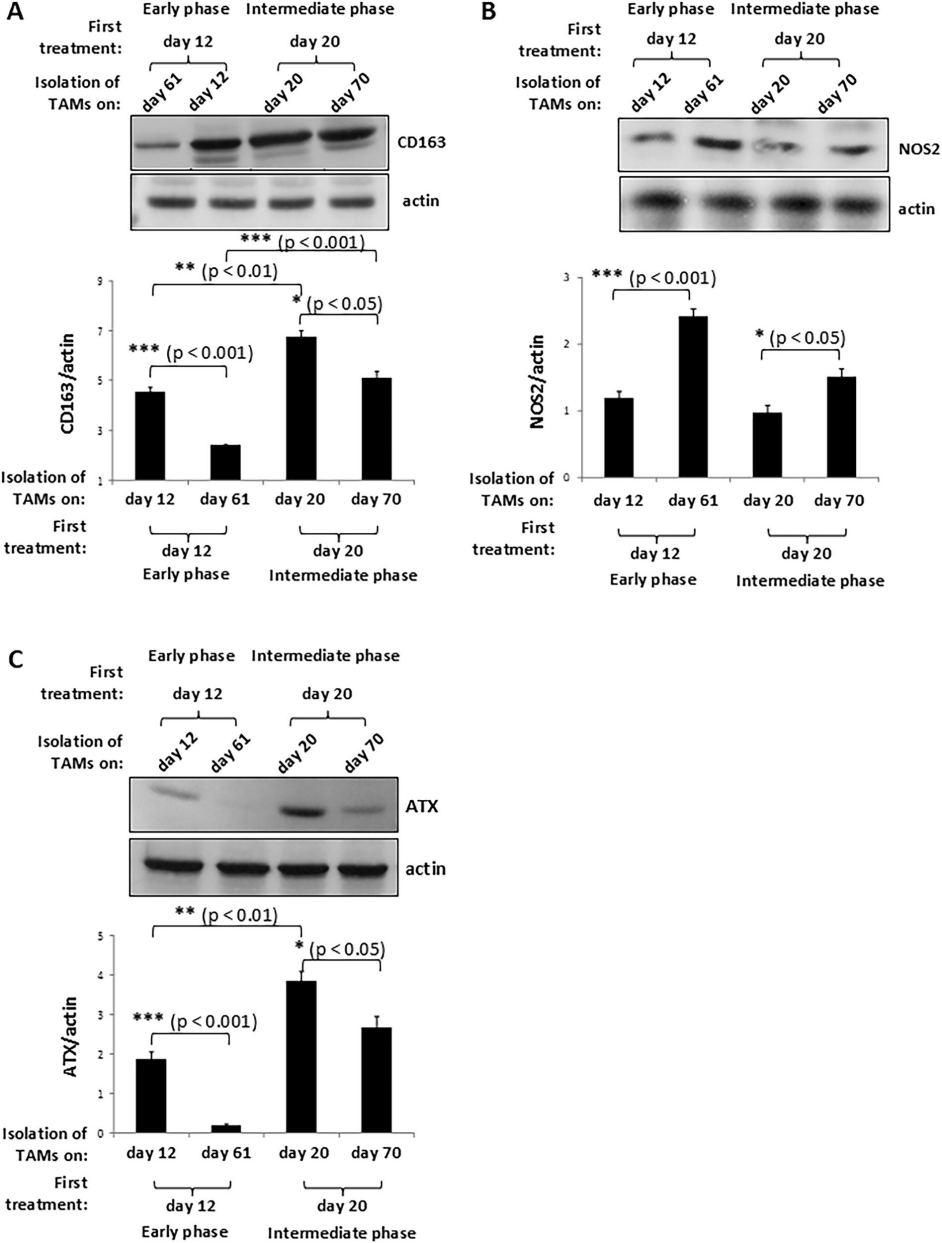
Impact of IOA-244 on the amount of CD163+ and NOS2+ macrophages and on TAMs-expressed ATX. TAMs were isolated from MDA-MB-231 breast early developed or established tumours harvested from BALB/c nude mice on day +12 (the day of the first treatment with IOA-244) and on day that the experiment ended (day +61) or from MDA-MB-231 breast tumours harvested from BALB/c nude mice on day +20 (the day of the first treatment with IOA-244) and on day that the experiment ended (day +70) respectively. The TAMs fraction was immunoblotted with an anti-CD163 (**A**), anti-NOS2 (**B**) or anti-ATX (**C**) specific antibody and the respective amounts of CD163 + , NOS2+ macrophages and ATX were calculated. *n* = 7–8 mice/group. All graphs represent means ± s.e.m. Statistically significant differences are indicated by *(*P* < 0.05), **(*P* < 0.01)or *** (*P* < 0.001), as determined by the Mann-Whitney test.

Balb/c nude mice lack T cells but produce macrophages. We thus next evaluated the abundance of CD163+ macrophages in tumours of MDA-MB-231 tumour-bearing Balb/c nude mice in which the treatment with IOA-244 started either when tumours were at an early phase or were established. To do this we isolated TAMs from excised early or intermediate phase tumours on the day of the first treatment with IOA-244 and on day that the experiment ended under both treatment conditions. Immunoblotting of TAMs fraction with an anti-CD163 specific antibody revealed that the abundance of CD163+ macrophages was drastically reduced on day that the experiment ended (day +61) in mice in which the inhibitor was administered *per os* in early phase tumours compared with CD163+ macrophages expressed on tumour environment on the first treatment day (day +12) (Fig. 4A). In contrast, only a modest IOA-244-induced decrease of CD163+macrophages was observed on day that the experiment ended (day +70) in mice in which the inhibitor was administered *per os* in intermediate phase tumours compared with CD163+macrophages expressed on tumour environment on the first treatment day (day +20) (Fig. 4A). Furthermore, the amount of CD163+macrophages in untreated tumours on day +20 (intermediate phase tumours) was significantly increased compared with that in untreated tumours on day +12 (early phase tumours) (Fig. 4A). Moreover, the amount of CD163+ macrophages on day +70 in tumours from mice receiving IOA-244 form day +20 (intermediate phase tumours) was significantly increased compared with that on day +61 in tumours from mice receiving IOA-244 form day +12 (early phase tumours) (Fig. 4A). We then assessed the impact of IOA-244 on the abundance of M1-like macrophages in tumour sites by immunoblotting the TAMs fraction with an anti-NOS2 specific antibody. NOS2 (or nitric oxide synthase (iNOS)) is a pro-inflammatory marker which characterises the M1 phenotype of TAMs and reduction of NOS2+ TAMs is correlated with tumour aggressiveness [70–72]. Interestingly, a significant increase in the expression levels of the M1 macrophage marker NOS2 was displayed in TAMs fraction isolated from excised tumours on day that the experiment ended (day +61) compared to that in TAMs fraction isolated from tumours on the first treatment day in mice receiving IOA-244 form day +12 (early phase tumours) (Fig. 4B). However, the respective increase of the amount of NOS2-expressing TAMs on day that the experiment ended (day +70) in mice receiving IOA-244 form day +20 (intermediate phase tumours) was less pronounced (Fig. 4B) which is in line with the respective less decrease of CD163 levels under the same experimental conditions (Fig. 4A).

These results indicate that the inhibition of p110δ PI3K by the IOA-244 beyond than prevented the accumulation of macrophages to tumour sites and the growth of TAMs under both conditions, IOA-244 blocked the transition of macrophages to M2-like phenotype and/or induced the transition of M2-like macrophages to M1-like phenotype or prevented the transition of M1- to M2-like phenotype when given at the earlier tumour phase. However, IOA-244 most likely was not able to confront the elevated amount of M2-like macrophages when treatment started at the established tumours.

We then explored whether IOA-244 has an impact on the expression of ATX by TAMs on early developed tumours and on established breast tumours. Interestingly, we found that the expression levels of ATX in the TAMs fraction of breast tumours (Fig. 4C) followed the same pattern with that of the amount of CD163+ macrophages under the same conditions (Fig. 4A). The expression levels of ATX were significantly reduced on the last day (day +61) of the experiment compared with those measured on the first treatment day only when the treatment with IOA-244 started early (day +12) after cancer cells inoculation (Fig. 4C). In contrast, the respective ATX expression levels on the last day (day +70) of the experiment were only slightly decreased when the treatment with IOA-244 started on established-intermediate phase tumours (day +20) (Fig. 4C). The expression of ATX was also found to be elevated in the TAMs fraction of untreated tumours on day +20 (intermediate phase) compared with that in untreated tumours on day +12 (early phase) (Fig. 4C) indicating a direct correlation between the amount of the M2-like macrophages and ATX on tumour sites of breast cancer.

Together, these data indicate that the IOA-244 targets the M2-like pro-tumorigenic macrophages and consequently the expression of ATX, leading to blockade of early developed breast tumours. In contrast, the effect of IOA-244 is diminished in established tumours that express highly elevated levels of CD163+ macrophages and ATX, underscoring the importance of timing in therapeutic intervention with IOA-244.

### Combined targeting of p110δ PI3K and ATX blocks the progression of established breast tumours

**Fig. 5:**
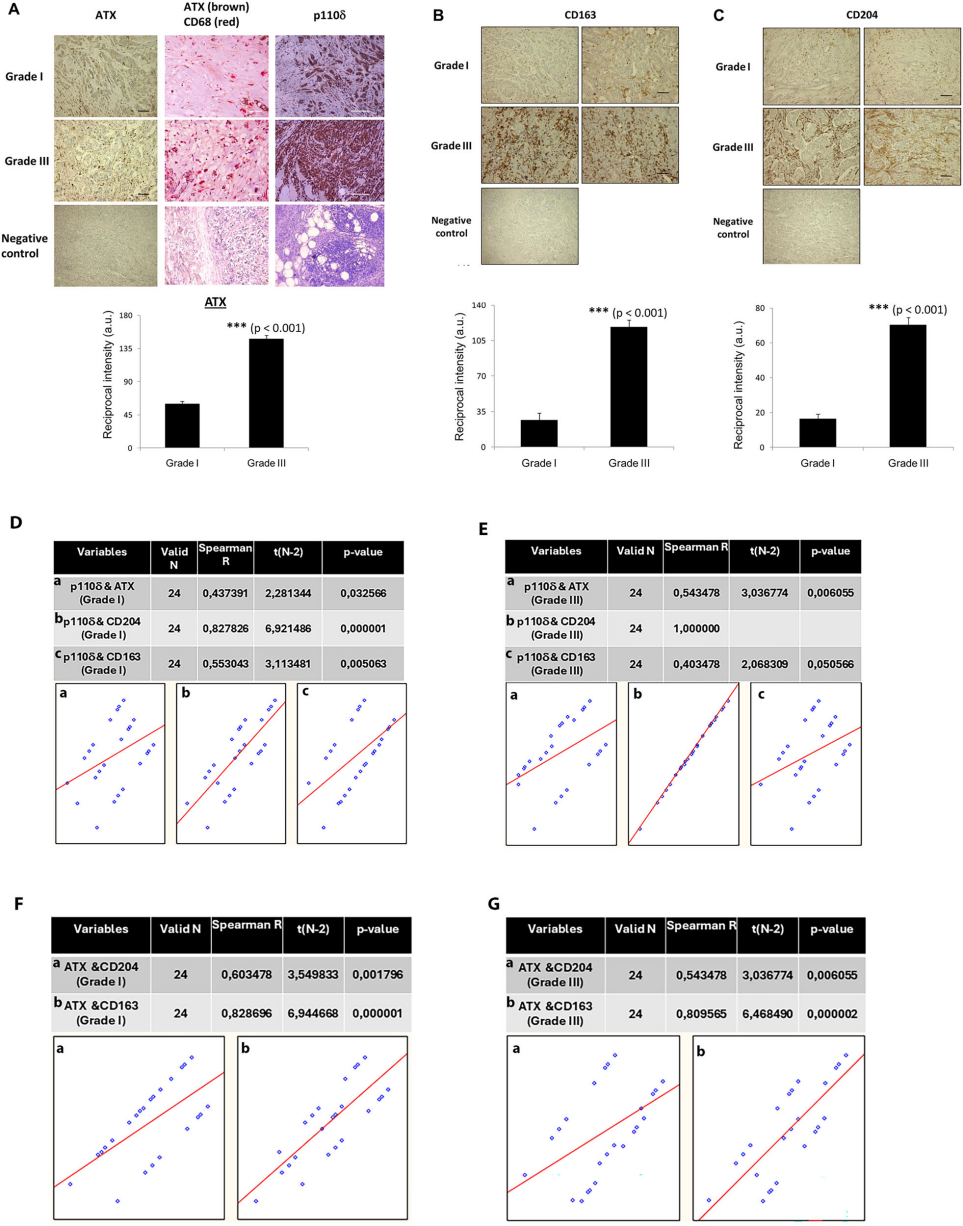
Impact of human breast tumour progression on expression levels of ATX, p110δ PI3K, CD163+ and CD204+ macrophages. **A** Representative images of human breast cancer tissue sections of grade I and grade III tumours stained with either, anti-ATX antibody (brown) and Hematoxylin (blue) (left column, scale bar = 100 μm), or double stained with anti-ATX (brown) and anti-CD68 (red) (middle column, scale bar = 50 μm) or anti-p110δ (brown) and Hematoxylin (blue) (right column, scale bar = 100 μm). In negative control sections each antibody was substituted by the respective IgG. The reciprocal intensity of the ATX−stained cells was determined and compared between tumours of grade I and III. (Means ± SD). **B** Representative images of human breast cancer tissue sections of grade I and grade III tumours stained with anti-CD163 antibody (brown) and Hematoxylin (blue). In negative control sections the anti-CD163 antibody was substituted by mouse IgG. Scale bar = 100 μm. The reciprocal intensity of the CD163−stained cells was determined and compared between tumours of grade I and III. (Means ± SD). **C** Representative images of human breast cancer tissue sections of grade I and grade III tumours stained with anti-CD204 antibody (brown) and Hematoxylin (blue). In negative control sections, the anti-CD204 antibody was substituted by mouse IgG. Scale bar = 100 μm. The reciprocal intensity of the CD204−stained cells was determined and compared between tumours of grade I and III. (Means ± SD). Statistically significant differences are indicated by *** (*P* < 0.001), as determined by the Mann-Whitney test. **D–G** Representative plots of Spearman’s correlation analysis of the relationships between p110δ-ATX (**Da, Ea**), p110δ-CD204 (**Db, Eb**), p110δ-CD163 (**Dc, Ec**), ATX-CD204 (**Fa, Ga**) and ATX-CD163 (**Fb, Gb**) expression in human tumour specimens of grade I and grade III, respectively.

We next sought to explore whether the differences in the amount of M2-like macrophages and the expression of ATX that we found between the untreated early developed and established MDA-MB-231 tumours are also observed in human breast cancers of different grades reflecting a progression of breast cancer from an early phase to a more established tumour. To evaluate this, we analysed the expression of ATX, CD163+ and CD204+ macrophages and the expression of ATX by macrophages and we also confirmed the expression of p110δ by immunohistochemistry in a collection of human breast carcinomas of grade I (*n* = 16) and grade III (*n* = 16) (Fig. 5). The expression of ATX in both, cancer tissues and macrophages in these tissues as it was confirmed by double immunostaining for ATX and CD68, as well as the expression of p110δ were found much higher in human breast carcinomas of grade III compared to that in grade I carcinomas (Fig. 5A). Furthermore, the staining of CD163+ (Fig. 5B) and CD204+ (Fig. 5C) was much stronger in grade III carcinomas compared to grade I carcinomas. The M2-like macrophages and ATX were detected mainly in the connective tissue cells surrounding cancer cells whereas p110δ was mainly detected as cytoplasmic staining in all carcinomas (Fig. 5). The collection of the human breast carcinomas used for the evaluation of ATX, p110δ, CD163+ and CD204+ macrophages was consisting of ER- and/or PR- positive as well as ER-and/or PR-negative tumours whereas all tumours were HER2-negative. The results were similar in all samples regardless of ER or PR assignment. To further explore the associations among the expression of p110δ, ATX, CD204 and CD163, we examined the relationships between p110δ-ATX, p110δ-CD204, p110δ-CD163, ATX-CD204 and ATX-CD163 expression in human tumour specimens of grade I and grade III (Fig. 5). Notably, Spearman’s correlation analysis revealed a significant positive correlation between all those relationships including a moderate correlation between p110δ-ATX in grade I (*r* = 0.437, *p* = 0.032) (Fig. 5Da) and grade III (*r* = 0.543, *p* = 0.006) (Fig. 5Ea) tumours, a very strong correlation between p110δ-CD204 in grade I (*r* = 0.828, *p* = 0.000001) (Fig. 5Db) as well as in grade III (*r* = 1) (Fig. 5Eb) tumours and a moderate correlation between p110δ-CD163 in grade I (*r* = 0.553, *p* = 0.005) (Fig. 5Dc) and grade III (*r* = 0.403, *p* = 0.05) (Fig. 5Ec) tumours. A moderate to strong correlation was also revealed between ATX-CD204 expression in grade I (*r* = 0.603, *p* = 0.0018) (Fig. 5Fa) and grade III (*r* = 0.543, *p* = 0.006) (Fig. 5Ga) whereas the correlation between ATX-CD163 expression was determined to be very strong in both, grade I (*r* = 0.829, *p* = 0.000001) (Fig. 5Fb) and grade III (*r* = 0.809, *p* = 0.000002) (Fig. 5Gb) tumours.

**Fig. 6:**
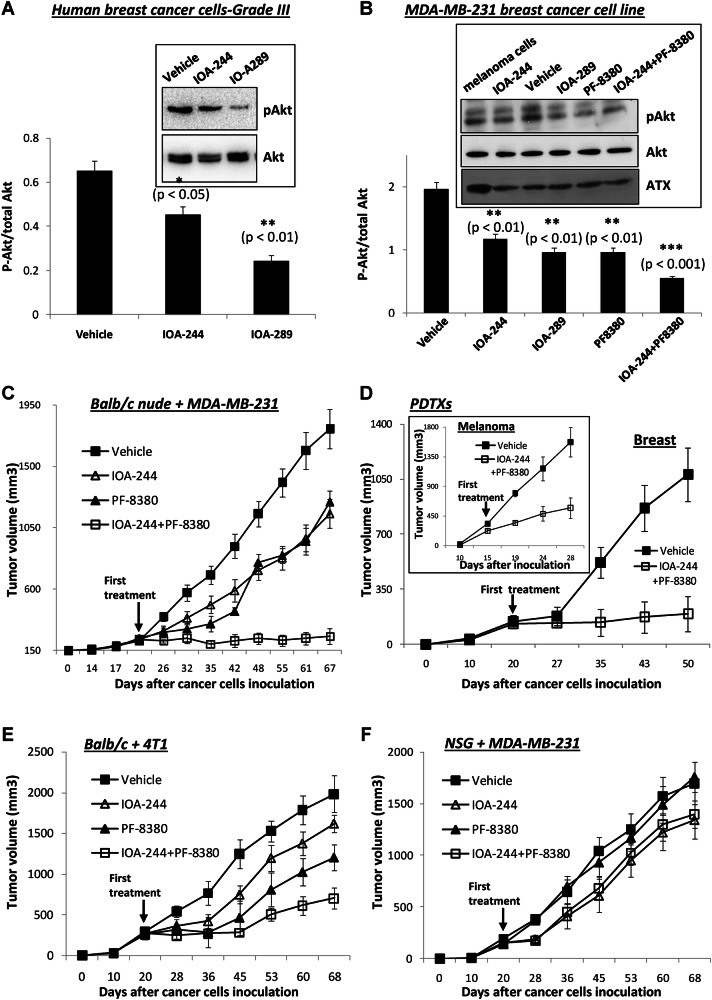
Combined treatment of established breast tumours with the IOA-244 p110δ inhibitor and PF-8380 ATX inhibitor blocks tumour growth. **A** Cancer cells were isolated from human breast cancer specimens of grade III and then treated with 10 μM of the IOA-244 p110δ inhibitor or IOA-289 ATX inhibitor for 1 h. Akt phosphorylation (on S473) was assessed by Western blotting of total cell lysates. **B** MDA-MB-231 cells were pretreated with 10 μM of the IOA-244 p110δ inhibitor or IOA-289 ATX inhibitor or PF-8380 ATX inhibitor or a combination of IOA-244 and PF-8380 and Akt phosphorylation (on S473) and the expression of ATX were assessed by Western blotting of total cell lysates. The 451Lu human melanoma cells were used as positive control for ATX expression. **C** BALB/c nude mice were inoculated with MDA-MB-231 breast cancer cells in the breast fat pad on day 0 and treated twice daily *per os* with vehicle or IOA-244 (30 mg/kg), or PF-8380 (30 mg/kg) or a combination of IOA-244 and PF-8380 from day +20. The tumours were measured by digital callipers and expressed as tumour volume. *n* = 7 mice/group. **D** Breast or melanoma (*inset*) PDTXs were categorised into the two indicated treatment groups (*n* = 3 mice/group) and were treated during the third passage of tumours. The results of two independent treatment groups were combined. **Ε** BALB/c mice were inoculated with 4Τ1 breast cancer cells in the breast fat pad on day 0 and treated twice daily *per os* with vehicle or IOA-244 (30 mg/kg), or PF-8380 (30 mg/kg) or a combination of IOA-244 and PF-8380 from day +20. The tumours were measured by digital callipers and expressed as tumour volume. *n* = 6–7 mice/group. **F** NSG mice were inoculated with MDA-MB-231 breast cancer cells in the breast fat pad on day 0 and treated twice daily *per os* with vehicle or IOA-244 (30 mg/kg), or PF-8380 (30 mg/kg) or a combination of IOA-244 and PF-8380 from day +20. The tumours were measured by digital callipers and expressed as tumour volume. *n* = 6–7 mice/group. All graphs represent means±s.e.m. Statistically significant differences are indicated by *(*P* < 0.05), **(*P* < 0.01) or ***(*P* < 0.001) as determined by the Mann-Whitney test.

We next assessed a potential impact of p110δ PI3K or ATX inhibition on the survival of cancer cells isolated from grade III human breast tumour specimens. IOA-244 reduced the phosphorylation levels of Akt in primary human breast cancer cells (Fig. 6A) however, IOA-289, a potent ATX-selective inhibitor [73], showed a more pronounced effect on the phosphorylated Akt (Fig. 6A). Although the expression of ATX by breast cancer cells is low at least compared to that by melanoma cells ([62, 63] and Fig. 6B), IOA-289 or PF-8380, another ATX specific inhibitor [74], significantly reduced the phosphorylation levels of Akt in MDA-MB-231 cells (Fig. 6B) and moreover, a combination of the IOA-244 p110δ inhibitor and PF8380 ATX inhibitor induced an even stronger reduction in Akt phosphorylation levels (Fig. 6B) indicating that a combination of p110δ PI3K and ATX inhibition might be effective in blocking the progression even of established breast tumours in vivo. Given the high expression levels of ATX by the tumour surrounding stroma [40, 46, 48] its inhibition will potentially have a strong impact on established tumours. To assess this, we evaluated the impact of the IOA-244 p110δ inhibitor, the PF-8380 ATX inhibitor, their combination or vehicle on tumour growth in MDA-MB-231-bearing Balb/c nude mice in which the treatment with the inhibitor(s) started on day +20 (Fig. 6C). PF-8380 is a small molecule specific inhibitor of ATX [74] with subnanomolar potency, good oral availability [74] and no toxic effects on mice [75, 76] and oral gavages of 30 mg/kg was found to cause a reduction in LPA levels in plasma and inflammatory tissue sites by more than 95% [74]. Interestingly, although IOA-244 or PF-8380 alone decreased but not blocked tumour growth rate, the combination of both inhibitors led, strinkingly, to an almost complete blockade of established breast tumour growth compared with mice receiving vehicle (Fig. 6C). Neither of the inhibitors alone nor the combined treatment with both, IOA-244 and PF-8380 affected the body weight of mice during the course of the experiments (Supplementary Fig. 2A). The activity of ATX in plasma from mice receiving the PF-8380 inhibitor was found to be decreased compared to that from mice receiving the vehicle (Supplementary Fig. 2B) confirming the effect of PF-8380 on ATX and indicating that the production of ATX by tumour-associated cells is also affected which might additionally account for the strong effect on tumour growth. The blockade of tumour growth is also reflected to a significant decrease in the BrdU-positive cells (Supplementary Fig. 2C) and a strong increase in the number of TUNEL-positive cells (Supplementary Fig. 2D) in tumour specimens from mice receiving both inhibitors, IOA-244 and PF-8380, compared with mice receiving vehicle indicating that the proliferative rate of tumours was prevented whereas apoptosis in tumour cells was induced. It is of note that in a limited number of PDTXs models that were developed by the implantation of grade III human breast cancer specimens (expressing high p110δ levels), following surgical removal from patients’ tumour, into a Balb/c nude mouse, the combined treatment of IOA-244 and PF-8380 also abolished breast tumour growth (Fig. 6D). In contrast, the combination of IOA-244 with PF-8380 regressed but not blocked the tumour burden of melanoma PDTXs (Fig. 6D, inset) in which melanoma tumours even though express high levels of ATX [62, 63] express low levels of p110δ compared to breast cancer cells and this is also in line with our previous findings showing that inhibition of p110δ in melanoma bearing mice has a valuable impact only in macrophages not in tumour cells [36, 77]. Because Balb/c nude mice produce macrophages but lack T cells, we further evaluated the impact of IOA-244, PF-8380 or their combination on tumour growth in two additional combinations of host mice and tumour cells. In particular, we assessed a) the tumour growth on the syngeneic model of 4T1 tumours which do not express p110δ in the Balb/c strain mice which have an intact immune system with normal macrophages and T cells (Fig. 6E) and b) the growth of MDA-MB-231 tumours which express p110δ in NOD *scid* gamma (NSG) mice that have defective macrophages and lack T cells (Fig. 6F). Oral administration of IOA-244 from day +20 either in 4T1 tumour-bearing Balb/c mice (Fig. 6E) or in MDA-MB-231-tumour bearing NSG mice (Fig. 6F) only modestly reduced tumour growth in both cases which is in line with the results described above showing an impact of IOA-244 on both, cancer cells and macrophages. On the other hand, the effect of PF-8380 in tumour progression in 4T1 tumour-bearing Balb/c mice (Fig. 6E) was similar with that observed in MDA-MB-231-bearing Balb/c nude mice (Fig. 6C), however, PF-8380 did not affect tumour growth in MDA-MB-231-tumour-bearing NSG mice (Fig. 6F), confirming the impact of macrophage-produced ATX on breast tumour growth. Moreover, the combination of IOA-244 with PF-8380 had a modest effect on tumour growth, equal with that of IOA-244 alone, in MDA-MB-231-tumour bearing NSG mice (Fig. 6F) whereas reduced but did not entirely block tumour growth in 4T1 tumour-bearing Balb/c mice (Fig. 6E). Blockade of tumour growth was only observed upon combination treatment of MDA-MB-231 (Fig. 6C) or breast patient derived (Fig. 6D) tumour bearing Balb/c nude mice which, even though lack T cells, have normal macrophages and p110δ-expressing tumours.

Together, these data indicate that IOA-244 acts on both, cancer cells and macrophages and moreover that macrophages play a critical role in the efficient impact of the combined treatment with IOA-244 and PF-8380 on preventing the progression of established breast tumours.

Overall, the above data strongly suggest that the IOA-244 p110δ-selective inhibitor as a single-agent treatment is effective in blocking early-phase breast tumours by exerting a strong effect in the polarisation of macrophages, whereas its combination with an ATX inhibitor is required to slow the growth rate of established breast tumours in mice (Supplementary Fig. 3).

## Discussion

Our work shows that the IOA-244 p110δ-selective inhibitor exerts a strong effect as a single agent treatment on breast cancer progression, correlating with a reduction in the abundance of CD163+ macrophages and a concomitant increase of NOS2+ macrophages and a reduction in the expression of ATX by TAMs in early phase breast tumours resulting in an almost total blockade of tumour growth in mice. We further demonstrated that the CD163+ macrophages and the expression of ATX were highly increased in established breast tumours compared with that found in early phase tumours in tumour-bearing mice and this pattern was also confirmed in human breast tumour specimens since the amount of CD163+ and CD204+ macrophages and ATX was much higher in human breast cancers of grade III compared with those of grade I. When tumours are already established, IOA-244 as a single agent treatment was inadequate to control the increase of M2-like macrophages and the expression of ATX. However, in combination with an inhibitor of ATX, they abolished the progression of established breast tumours.

In human tissues, macrophages can be divided into three major subpopulations, namely naïve or M0 macrophages, which are derived from the bone marrow, pro-inflammatory or antitumor M1-like macrophages and anti-inflammatory or protumour M2-like macrophages [78–80]. The differentiation and polarisation of macrophages, independently of their origin, to pro- or anti-inflammatory macrophages is controlled by the extracellular microenvironment and the cytokines present in this microenvironment [79, 81]. One of the subtypes of anti-inflammatory or M2-like macrophages that are found in cancers is commonly known as tumour-associated macrophages (TAMs) [78]. M2-like TAMs are among the cell types of tumour microenvironment with the strongest influence on tumour progression by producing anti-inflammatory and immunosuppressive cytokines [82, 83] and pro-angiogenic factors [83] promoting the suppression of anti-cancer immune responses, tumour vascularisation, invasiveness and metastasis and are associated with poor response to therapy [66, 84]. M2-like TAMs overexpress CD163 (macrophage scavenger receptor-B), CD204 (macrophage scavenger receptor-A) and CD206 (mannose receptor-1) [47, 85–87]. In breast cancer, various studies including clinical studies and experimental studies using human breast cancer samples, cell lines, and murine breast cancer models have shown that M2-like TAMs are the most prominent immune cells in the breast cancer microenvironment, constituting more than 50% of the cell mass and correlating with poor prognosis in 80% of the cases examined [64, 65, 88]. M2-like TAMs were also found to influence the surrounding tumour cells and the response to cancer therapies, including conventional cancer treatments such as chemotherapy and radiation therapy as well as immunotherapy and targeted therapy [66, 67, 89]. Especially in the late phases of breast cancer, TAMs were found to express very high levels of the CD163 antigen due to the presence of the tumour [86] having a strong anti-inflammatory activity [90] leading thus to worse clinical outcome of the patients [91].

Furthermore, M2-like TAMs are considered to be functionally involved in the production of ATX and LPA directly or indirectly by producing inflammatory mediators that stimulate tumour stroma cells to increase ATX production [46, 68, 92]. In contrast to TAMs, breast cancer cells express very low levels of ATX [46, 47], with the main source of ATX and LPA in breast tumours being the tumour-associated stroma [40, 46, 48]. Specifically, CD163 + CD206+ TAMs have been found to play an essential role as main producers of ATX [68] and CD163+ macrophages show a high stromal ATX positivity in breast tumours with adipose tissue-rich stroma [47, 93]. It is of note that the IOA-289 ATX selective inhibitor suppressed the growth of E0771 breast tumours in mice in which ATX was knocked out (KO) in adipocytes indicating that the source of ATX is other than adipocytes [94]. Therefore, our current results add further substances to the rationale that co-targeting M2-like TAMs and ATX in the tumour microenvironment will be a promising therapeutic strategy to block tumour growth or to improve the efficacy of breast cancer treatments.

Our present results clearly show that the IOA-244 p110δ-selective inhibitor strongly affects the progression of early developed breast tumours leading to an almost complete elimination of tumour growth whereas it also prevents the spontaneous intravasation of breast cancer cells. The effectiveness of IOA-244 in preventing breast tumour growth and metastasis is not only a result of its efficacy to inhibit the survival of cancer cells but also derives from the modulation of macrophage recruitment to tumour sites and especially their polarisation to pro-tumourigenic M2-like phenotype, which consequently leads to suppression of the expression of ATX. Pharmacological or genetic inactivation of PI3K p110δ has been previously shown to block chemotaxis of macrophages [95] and to affect their polarisation [96], whereas we have recently documented that the inactivation of p110δ in macrophages is sufficient to prevent the localisation of macrophages into tumour stroma and consequently to suppress tumour growth and metastasis [17]. In the present study, we found that IOA-244 prevents the accumulation of total macrophages to tumour sites independently on the timing of its first administration which most likely accounts for the equal blockade of metastasis under both conditions. Moreover, IOA-244 was able to eliminate the amount of CD163+ pro-tumourigenic M2-like macrophages and at the same time to increase the amount of NOS2+ anti-tumour M1-like macrophages, at least in early developed tumours.

The CD163+ macrophages originate either from extravasation of monocytes or from polarisation of already present M1 pro-inflammatory macrophages [87, 97]. It is well known that the polarisation of macrophages to the pro-tumourigenic M2 phenotype is mainly regulated by T cells, especially Th2 cells [87, 98], whereas regulatory T cells (Tregs) were also found to facilitate the M2-polarisation of macrophages [99–101]. On the other hand, inhibition of p110δ PI3K has been found to impact tumour growth by reducing the immune-suppressive function of Tregs [102]. However, in the present study IOA-244 abolished the amount of CD163+ macrophages despite the fact that Balb/c nude mice lack T cells, so those T-cell effects cannot explain its ability to reduce M2-like macrophage numbers. Therefore, the effect of p110δ inhibition by the IOA-244 in reducing the abundance of CD163+ macrophages to tumour sites has to be attributed mainly to a negative effect of IOA-244 per se on the polarisation of macrophages towards the M2-like phenotype. Given that IOA-244 increased, in contrast, the amount of M1-like macrophages it would be also possible IOA-244 to affect either negatively the transition of M1- towards M2-like phenotype or positively the transition of M2- towards M1-like phenotype. It is well known that the plasticity of M1 and M2 macrophages is high and thus macrophage phenotype can be converted into each other upon tumour microenvironment changes or therapeutic interventions and moreover, previously reported data have shown that the p110δ PI3K is involved in the expression of M2-macrophage markers in neonatal primary cardiomyocytes and murine macrophages [103]. Additionally, the dextran sodium sulfate (DSS) was found to induce colitis in p110δ PI3K-deficient mice and this was correlated with reduced numbers of arginase I + M2 macrophages in the colon [104]. In addition, IOA-244 could indirectly regulate the polarisation of macrophages by its effect to block B cells activity [33, 35]. A number of factors, including interleukin (IL)-6 and IL-10 regulate the M2 polarisation of macrophages [78, 79] and these cytokines are produced by B cells [87, 105]. Indeed, B cells were found to induce the polarisation of peritoneal macrophages and to reprogramme TAMs into an M2-like phenotype through secretion of IL-10 [106], whereas other evidence suggests that the regulatory B cells (Bregs) inhibit the immune responses through IL-10 production in breast tumour sites [107]. B cells were also found to promote the protumourigenic effects of macrophages through various mechanisms in several cell systems [87, 105, 108–110].

Our results indicate that IOA-224 is effective in eliminating the number of CD163+ macrophages and consequently the expression of ATX, resulting in blockade of early developed breast tumours. However, IOA-244 did not have the same effectiveness as a single agent treatment in established breast tumours expressing much higher levels of CD163+ macrophages and ATX compared to early developed tumours. It seems likely that a threshold in the abundance of CD163+ macrophages is essential for IOA-244 to achieve maximum effectiveness against breast tumour growth. In agreement with this line of thought, we found that the efficacy of oral treatment with IOA-244 depends on the timing of first administration and this inversely coincides with elevated amounts of both CD163+ macrophages and ATX on day of treatment onset. Moreover, in line with the known LPA (the product of ATX)-induced phosphorylation of ERK, IOA-244 had only a modest effect on pERK levels in established tumours expressing high levels of ATX, whereas in early developed tumours expressing low ATX, the decrease in pERK levels by IOA-244 was more pronounced. The fact that the use of IOA-244 as a combinatorial regimen together with an inhibitor of ATX totally blocked tumour burden growth in established tumours indicates that a key effector that mediates the high efficacy of IOA-244 is ATX.

IOA-244 is the first-in-class, highly selective and non-ATP-competitive p110δ PI3K inhibitor and is currently in clinical Phase I/II investigation in solid and haematological tumours [35]. Overall, our data herein point to IOA-244 as a potentially highly effective drug for breast cancer treatment which targets both breast cancer cells and pro-tumourigenic macrophages in cancer stroma. The present results also suggest that depending on the phase of the tumour and the respective M2-like macrophage presence and ATX expression, IOA-244 could, in the future, be used clinically either as a single agent or in combination with an ATX inhibitor for breast cancer treatment.

## Materials and methods

### Chemicals

The PI3K p110δ inhibitor IOA-244 was provided by iOnctura SA. IOA-244 was re-suspended in DMSO for in vitro experiments and in 30% PEG-400, 0.5% Tween-80 and 5% propylene glycol for experiments in mice. EGF was from Sigma (St. Louis, MO, USA). Antibodies to phosphor S473-Akt (#4060, RRID:AB_2315049), total Akt (#4691, RRID:AB_915783), caspase3 (#9662, RRID:AB_331439), β-actin (#4970, RRID:AB_2223172) were obtained from Cell Signalling Technology Inc. Other sources for antibodies were as follows: F4/80 (Bio-Rad #MCA497GA, RRID:AB_323806), ATX (MBL # D323-3, RRID:AB_2819353), vimentin (Thermoscientific #RM-9120, RRID:AB_722362), Ki-67 mAb (Pierce #MA5 -15690, RRID:AB_10979995).

### Isolation of cancer cells from human breast tumour specimens

Cancer cells were isolated from human breast tumour specimens following surgical resection of the primary tumour. All procedures were approved by the Board of Directors of the General University Hospital of Crete (Decision No. 987). Tissue dissociation was performed using an enzyme mixture containing 0.05 mg/mL Collagenase I, 0.05 mg/mL Collagenase IV, and 0.01 mg/mL DNase I in HBSS, followed by cancer cell isolation using the Cancer Cell Isolation Kit (Thermo Scientific LSG). Isolated cells were lysed either in a buffer containing 150 mM NaCl, 1.5 mM MgCl₂, 1 mM EGTA, 10% glycerol, 100 mM NaF, 25 mM glycerophosphate, 1% IPEGAL, 1 mM DTT, 10 mM sodium pyrophosphate, 1 mM PMSF, 10 μg/mL aprotinin, 10 mM Na₄VO₃, and 50 mM HEPES (pH 7.4) for PTEN lipid phosphatase activity assays, or in a buffer containing 20 mM Tris-HCl (pH 7.4), 137 mM NaCl, 1 mM CaCl₂, 1 mM MgCl₂, 1 mM sodium orthovanadate, 1% NP-40, and 1 mM PMSF. Lysates were subsequently clarified by centrifugation in a refrigerated microcentrifuge.

### Cell culture of cancer cell lines

The human breast cancer cell line MDA-MB-231 (RRID:CVCL_0062) was kindly provided by Bart Vanhaesebroeck (Ludwig Institute for Cancer Research, London, UK). Cells were cultured in DMEM (Invitrogen, Life Technologies) supplemented with 10% foetal calf serum (FCS) and 1% penicillin–streptomycin at 37 °C in a humidified atmosphere containing 5% CO₂. The murine breast cancer cell line 4T1 (RRID:CVCL_0125) was maintained in RPMI 1640 medium supplemented with 10% FCS, 2 mM L-glutamine, 1 mM sodium pyruvate, and 50 µg/ml penicillin–streptomycin. All cell lines were routinely tested for mycoplasma contamination. Prior to injection into mice, cells were cultured to approximately 70% confluence, harvested, counted, and resuspended in sterile PBS. Where indicated, hypoxic conditions were induced by incubating cells at 1% O₂, 5% CO₂, and 94% N₂ for 48 h before experimentation [111].

### Isolation of TAMs and cancer cells from tumours in mice

To ensure optimal purity of tumour-associated macrophages, tumours were excised and carefully dissected to remove lymph nodes, necrotic tissue, adipose tissue, and surrounding blood vessels. The remaining tumour tissue was placed in a Petri dish and enzymatically digested using a dissociation buffer consisting of RPMI 1640 medium supplemented with 5% FBS, collagenase/hyaluronidase, and DNase I (10 U/mL) [112]. TAMs were isolated following the procedure previously described [36–39, 49, 50, 112]. Isolation of MDA-MB-231 cancer cells from tumours was performed using the procedure described above for human breast tumour specimens.

### Western blotting analysis

For Western blot analysis of total cell lysates, 50–70 µg of protein per sample was resolved by SDS–PAGE and transferred onto PVDF membranes. Membranes were incubated with the indicated primary antibodies, followed by detection using enhanced chemiluminescence (GE Healthcare).

### Caspase-3/7 assay

Caspase-3/7 activities were assessed using the Caspase-Glo® 3/7 Assay (Promega) according to the instructions of the manufacturer.

### LDH assay

Cell toxicity was assessed by measurement of lactate dehydrogenase (LDH) released into the cell culture supernatant using an LDH assay kit (Promega), which assesses the membrane integrity of cells, as previously reported [113]. In brief, after treatment of cells with different concentrations of IOA-244 for 48 h, the cell culture medium was collected. Then, upon centrifugation, the supernatant was obtained and the level of LDH was measured using the LDH kit. The percentage of cytotoxicity was calculated using the formula: percent cytotoxicity =100 × (experimental sample-culture medium background)/(maximum LDH release - culture medium background).

### Mice

All mice were maintained in a pathogen-free facility at the Medical School of the University of Crete. Experimental procedures were approved by the Research Animal Care Committee of the Medical School, University of Crete, and by the Veterinary Department of Heraklion Prefecture (Protocol No. 269920), in accordance with national and EU regulations. Female BALB/c nude mice (RRID:IMSR_RJ:BALB-C-NUDE) were obtained from Charles River Laboratories.

Group sample sizes were determined based on calculations using the NC3Rs-recommended Resource Equation method, pilot experiments, and prior knowledge of the variability of breast tumours in BALB/c nude mice, NOD.*Cg-Prkdc*^*scid*^ *Il2rg*^*tm1Wjl*^ (NOD scid gamma or NSG; RRID:IMSR_JAX:005557), and BALB/c mice (RRID:IMSR_TAC:BALB). All mice used were female, age-matched (2–3 months), and in good health. Animals were randomly assigned to experimental groups at the start of each experiment. Following tumour cell injections, an equal number of mice were randomly allocated to each treatment arm. Each experiment included a minimum of 6–7 mice per group (specific numbers are indicated in the figure legends) and was independently repeated at least three times to assess reproducibility.

### In vivo studies of tumour growth in xenograft models

Female BALB/c nude mice, or, where indicated, NSG or BALB/c mice, were inoculated subcutaneously into the right mammary fat pad on day 0 with 1 × 10⁶ MDA-MB-231 or, where specified, 4T1 cells in 100 µL PBS. Mice were randomly assigned to a control group receiving vehicle or a treatment group receiving IOA-244 (30 mg/kg) by oral gavage twice daily, starting on day +12 or day +20. In separate experiments, the ATX inhibitor PF-8380 (30 mg/kg) was administered twice daily by oral gavage. [75, 76]. Tumour growth was monitored by measurement of the longest perpendicular tumour diameters using a digital calliper every 3–6 days. The tumour volume (*V*) was calculated using the formula *V* (mm^3^) = (length (mm) × width (mm)^2^) × 0.5 [114, 115]. Mouse body weight was monitored weekly. At the end of the study, animals were euthanized, and primary tumours and lungs were collected for H&E staining or immunohistochemistry, as described below.

### Mice bearing breast or melanoma patient-derived xenograft (PDX)

PDTXs were developed by the implantation of human breast or melanoma specimens, following surgical removal from respective patient’s tumour into an NSG mouse [116]. Patients gave their written informed consent and the procedures were approved by the Board of Directors of General University Hospital of Crete (Decision No 987). Briefly, the viable tumour was dissected into small pieces of about 3–8 mm^3^ prior to implantation and directly transplanted subcutaneously into a mouse, which was designated generation 0. Upon engraftment of tumours in the first cohort of recipient mice, the palpable growing tumours were removed and grafted onto another cohort (usually from 1 mouse to 3 mice) that was designated generation 1 and then serially over several passages. Experiments were conducted on the second or third generation [116]. PDTX mice were randomly divided into treatment groups (*n* = 3 mice per group). The treatment conditions and tumour burden monitoring were performed as described above.

### Tumour cell blood burden

The tumour cell blood burden was assessed as previously described [61]. Mice were anaesthetised, and blood was collected from the right atrium using a heparin-coated 25-gauge syringe. Samples were plated in culture dishes with Geneticin to selectively grow tumour cells, and the blood burden was calculated as the number of colonies per volume of blood.

### Measurement of ATX activity

The blood was collected as described above, and the measurement of ATX activity in 10 μl of plasma was performed as previously described [36, 77]. The activity of ATX represents the choline released from LPC divided by the volume of the plasma taken [77].

### Immunohistochemistry

Paraffin-embedded, formalin-fixed mouse tissues were processed as previously described [117]. In brief, tissue sections were deparaffinized and rehydrated through a graded ethanol series. Endogenous peroxidase activity was quenched by incubation with 3% hydrogen peroxide for 30 min at 20 °C, followed by rinsing in PBS. Antigen retrieval was performed either by digestion with 0.2% trypsin for 10 min at room temperature (for the anti-pAkt antibody) or by heat-mediated retrieval in citrate buffer (pH 6.0) for 40 min (for the anti-vimentin antibody). Sections were then blocked to prevent nonspecific binding using goat serum (for pAkt staining) or 1% BSA for 1 h at room temperature (for the other antibodies), and subsequently incubated with primary antibodies overnight at 4 °C (pAkt, 1:50; Cell Signaling #4060; F4/80, 1:50; Bio-Rad #MCA497GA; vimentin, 1:200; Thermo Scientific #RM-9120). After washing, sections were incubated with HRP-conjugated anti-rat secondary antibody (for F4/80) or HRP-conjugated anti-rabbit secondary antibody (for pAkt and vimentin), developed using DAB and H₂O₂, counterstained with hematoxylin, and mounted with Vectashield mounting medium (Vector Laboratories). For human cancer specimens, immunohistochemical analyses were performed on 4-µm-thick sections of formalin-fixed, paraffin-embedded archival breast cancer tissues obtained from the Department of Pathology, University Hospital of Heraklion, Greece, or from breast tumour samples collected following surgical resection of the primary tumour, with approval from the Board of Directors of the General University Hospital of Crete (Decision No. 987). After deparaffinization and rehydration, sections were subjected to antigen retrieval by microwave heating in EDTA buffer (pH 8.0) at 500 W for 15 min, followed by cooling at room temperature for 20 min. Tissue sections were then incubated with primary antibodies either overnight at 4 °C—ATX (rat, clone 4F1, 1:40; MBL Life Science, #D323-3) and CD163 (mouse, clone 10D6, 1:50; Thermo Fisher Scientific, #MA5-11458, RRID:AB_10982556)—or for 1 h at room temperature when anti-CD204 (clone J5HTR3, 1:200; Thermo Fisher Scientific, #14-9054-82, RRID:AB_2662676) or anti-p110δ (1:200; Abcam #ab200372, RRID:AB_3674611) antibodies were used. Visualisation was performed using the DAKO REAL EnVision Detection System (K5007) with DAB chromogen, in accordance with the manufacturer’s instructions. For double immunostaining of ATX and CD68, sections were subsequently incubated with the anti-CD68 antibody (Thermo Scientific #MA5-13324, RRID:AB_10987212) for 1 h at room temperature (1:200 dilution), followed by detection using the EnVision G2 System and alkaline phosphatase substrate (Permanent Red), as per the manufacturer’s protocol. Counterstaining was carried out with Mayer’s hematoxylin. Quantification of positive cells and reciprocal intensity measurements [118] was performed using ImageJ software (NIH; RRID:SCR_003070) or Adobe Photoshop 2020 (RRID:SCR_014199). Data are presented as mean ± s.e.m. (standard error of the mean) from 5 to 8 randomly selected fields per section and three sections per determination.

### BrdU incorporation

To assess the proliferative activity of tumour cells, a BrdU staining kit (Millipore #2760) was used following the manufacturer’s protocol. In brief, mice were administered 100 mg/kg body weight of 5-bromo-2′-deoxyuridine (BrdU; Calbiochem) via intraperitoneal injection 2 h prior to euthanasia, and BrdU-positive tumour cells were subsequently detected. The quantification of BrdU-positive cells was performed using ImageJ software (NIH). Data are expressed as means ± s.e.m. of BrdU-positive cells relative to hematoxylin-stained cells, counted from 5 to 8 randomly selected fields per section across 3 sections per measurement. BrdU incorporation assays were conducted on tissues from at least three independent experimental groups for each treatment condition, with consistent results observed across replicates.

### TUNEL assay

Apoptosis in tumour cells was detected using the DeadEnd Colorimetric TUNEL System (Promega #G7130) according to the manufacturer’s instructions. TUNEL-positive cells were quantified using ImageJ software (NIH). Data are expressed as means ± s.e.m. of TUNEL-positive cells relative to hematoxylin-stained cells, counted from 5 to 8 randomly selected fields per section across three sections per measurement. The TUNEL assay was performed on tissues from at least three independent experimental groups of animals for each treatment condition, and comparable results were obtained across experiments.

### Statistical analysis

Error bars displayed in the Figure section represent SEM or SD and were calculated from technical or biological replicates as described in the figure legends and in the description of the respective methods. The data shown are representative of at least three independent experiments, including animal studies, histological images, blots and gels. Data were analysed using the STATISTICA 7 statistical software package (RRID:SCR_014213). Statistical significance was determined using the non-parametric Mann-Whitney test; *P < 0.05; **P < 0.01; ***P < 0.001. Spearman’s rank correlation was used to assess the relationship between two variables using Statistica 64 software, with statistical significance set at *p* < 0.05.

## Supplementary information

SUPP FIGURE Legends

Supp Fig1

Supp Fig2

Supp Fig3

Supp Fig4

## Data Availability

The authors declare that the data supporting the findings of this study are available within the paper and/or are available upon request from the corresponding author. Full and uncropped western blots are shown in Supplementary Fig. 4.
