# Supplementary material for: IOA-244, a novel p110δ PI3K inhibitor, blocks breast tumour progression on either mono- or combined-therapy
Source: Cell Death Discov. 2026 Mar 27;12:229. doi: 10.1038/s41420-026-03073-3 (PMC13184035; doi:10.1038/s41420-026-03073-3)
Supplement: Supplementary file 1 — SUPP FIGURE Legends [file 41420_2026_3073_MOESM1_ESM.docx]

**SUPPLEMENTARY FIGURE LEGENDS**

**Supplementary Figure 1**/ **Impact of IOA-244 p110δ−selective inhibitor on cell proliferation, apoptosis and toxicity in MDA-MB-231 breast cancer cells. A,** MDA-MB-231 cells were pretreated with the indicated concentrations of IOA-244, followed by stimulation with EGF (50ng/ml, 10 min) and then Akt phosphorylation (on S473) was assessed by Western blotting of total cell lysates. **B,** MDA-MB-231 cells were pretreated with the indicated concentrations of Idelalisib, followed by stimulation with EGF (50ng/ml, 10 min) and then Akt phosphorylation (on S473) was assessed by Western blotting of total cell lysates. **C,** MDA-MB-231 cells were treated with the indicated concentrations of IOA-244 and EGF (50ng/ml) for 48h and then cell proliferation was determined by assessing KI-67 by Western blotting of total cell lysates. **D,** MDA-MB-231 cells were treated with the indicated concentrations of IOA-244 and EGF (50ng/ml) for 48h and then apoptosis was determined by assessing pro-caspase-3 and cleaved-caspase-3 by Western blotting of total cell lysates. **E,** MDA-MB-231 cells were exposed to indicated concentrations of IOA-244 and EGF (50ng/ml) for 48 hours and apoptosis was detected by measuring caspase-3/7 activity. **F,** MDA-MB-231 cells were exposed to indicated concentrations of IOA-244 and EGF (50ng/ml) for 48 hours and cell toxicity was detected by measuring the LDH release. All graphs represent means±s.e.m. Statistically significant differences are indicated by * (P < 0.05) or *** (P < 0.001), as determined by the Mann-Whitney test.

**Supplementary Figure 2/The combined treatment of established breast tumours with the IOA-244 p110δ inhibitor and PF-8380 ATX inhibitor impacts on the proliferation and apoptosis of tumour cells without affecting the body weight of mice. A,** Body weight of mice during the course of the experiment. **B,** ATX activity in the plasma from mice which received the vehicle or PF-8380 (30 mg/kg). **C,** Cell proliferation in tumours excised from mice that were treated with the combination of IOA-244 and PF-8380 or vehicle was determined by BrdU incorporation (brown spots) followed by comparison of BrdU positive (BrdU+/Hem+) cells in the respective tumours. **C,** Apoptosis in tumours excised from mice that were treated with the combination of IOA-244 and PF-8380 or vehicle was determined by TUNEL assay (brown spots) followed by comparison of TUNEL- positive (TUNEL+/Hem+) cells in the respective tumours. All graphs represent means±s.e.m. Statistically significant differences are indicated by ***(P < 0.001) as determined by the Mann-Whitney test.

**Supplementary Figure 3/ Effect of IOA-244 on breast tumour cells and macrophages as a single agent in early phase tumours or in combination with an ATX inhibitor (PF-8380) in established tumours.** M2-like TAMs in breast tumour microenviroment produce ATX either directly or indirectly by producing inflammatory mediators (IMs) that stimulate tumour stroma cells to increase the production of ATX which then stimulates breast cancer cells to secrete cytokines that further mediate the production of ATX by the adjacent tissues. M2-like macrophages and TAMs-expressed ATX are at significantly higher levels in established tumours compared with that in early phase tumours. The IOA-244 p110δ-selective inhibitor affects equally the accumulation of macrophages to tumour sites and the growth of TAMs as well as the survival and proliferation of breast cancer cells, independently on the onset of its administration. When the treatment with IOA-244 starts on early phase tumours, IOA-244 is effective as a single agent treatment to reduce the number of the M2-like macrophages, either by blocking the transition of macrophages to M2-like phenotype or by inducing the transition of M2- to M1-like phenotype or by preventing the transition of M1- to M2-like phenotype leading to increased numbers of M1-like macrophages and to reduced expression of ATX by TAMs (left panel). IOA-244 as a single agent treatment is inadequate (dashed lines) to control the highly elevated expression of M2-like macrophages and ATX in established tumours however, in combination with an inhibitor of ATX (PF-8380) can achieve maximum effectiveness against the abundance of M2-like macrophages and the high expression levels of ATX (right panel).

**Supplementary Figure 4**/ Unedited images for Figure 1C,1D, Figure 2C,2D, Figure 4A,4B,4C, Figure 6A,6B.
