# Supplementary figures and images for: IOA-244, a novel p110δ PI3K inhibitor, blocks breast tumour progression on either mono- or combined-therapy
Source: Cell Death Discov. 2026 Mar 27;12:229. doi: 10.1038/s41420-026-03073-3 (PMC13184035; doi:10.1038/s41420-026-03073-3)

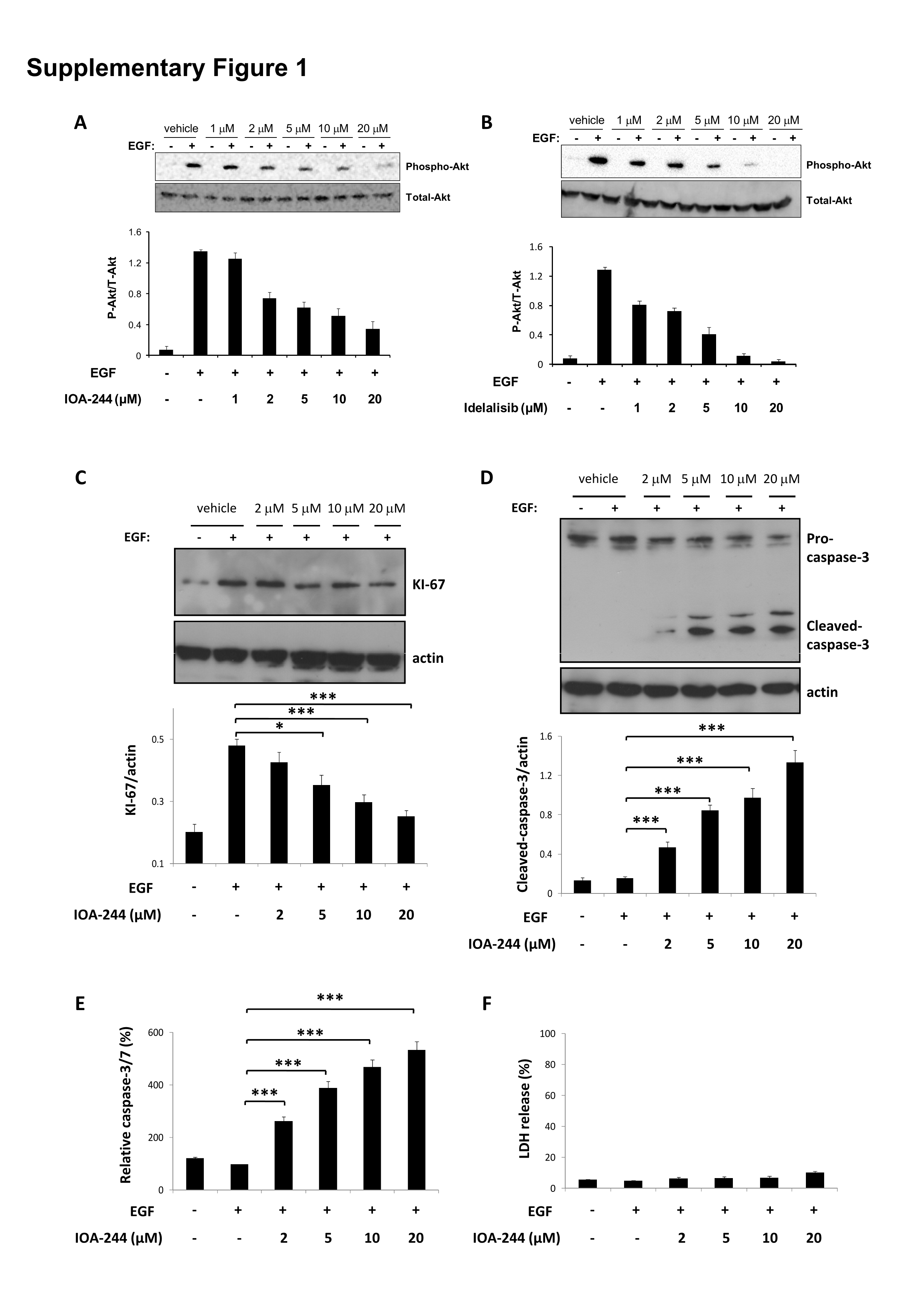

Supplement: Supplementary file 2 — Supp Fig1 [file 41420_2026_3073_MOESM2_ESM.tif]

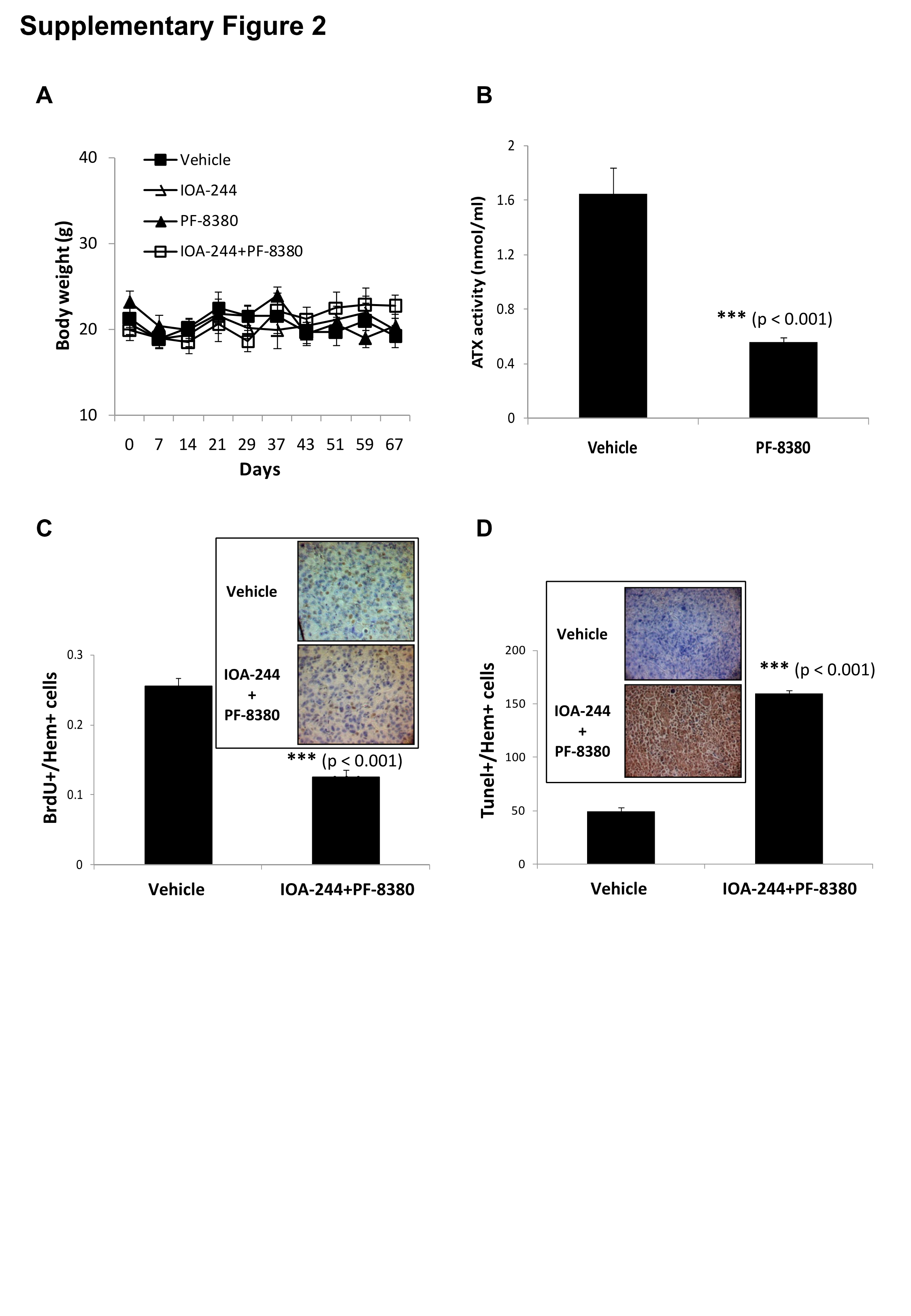

Supplement: Supplementary file 3 — Supp Fig2 [file 41420_2026_3073_MOESM3_ESM.tif]

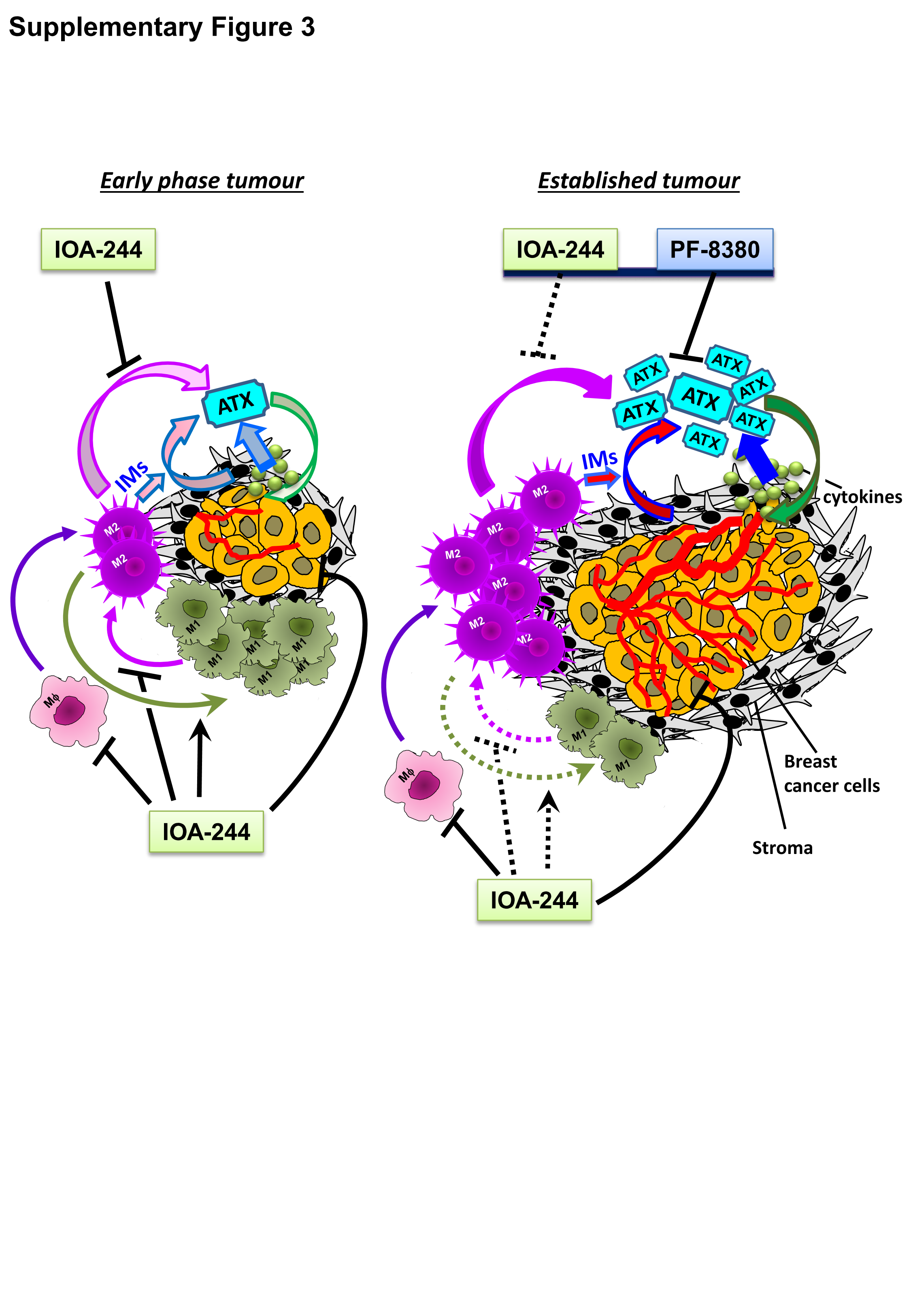

Supplement: Supplementary file 4 — Supp Fig3 [file 41420_2026_3073_MOESM4_ESM.tif]

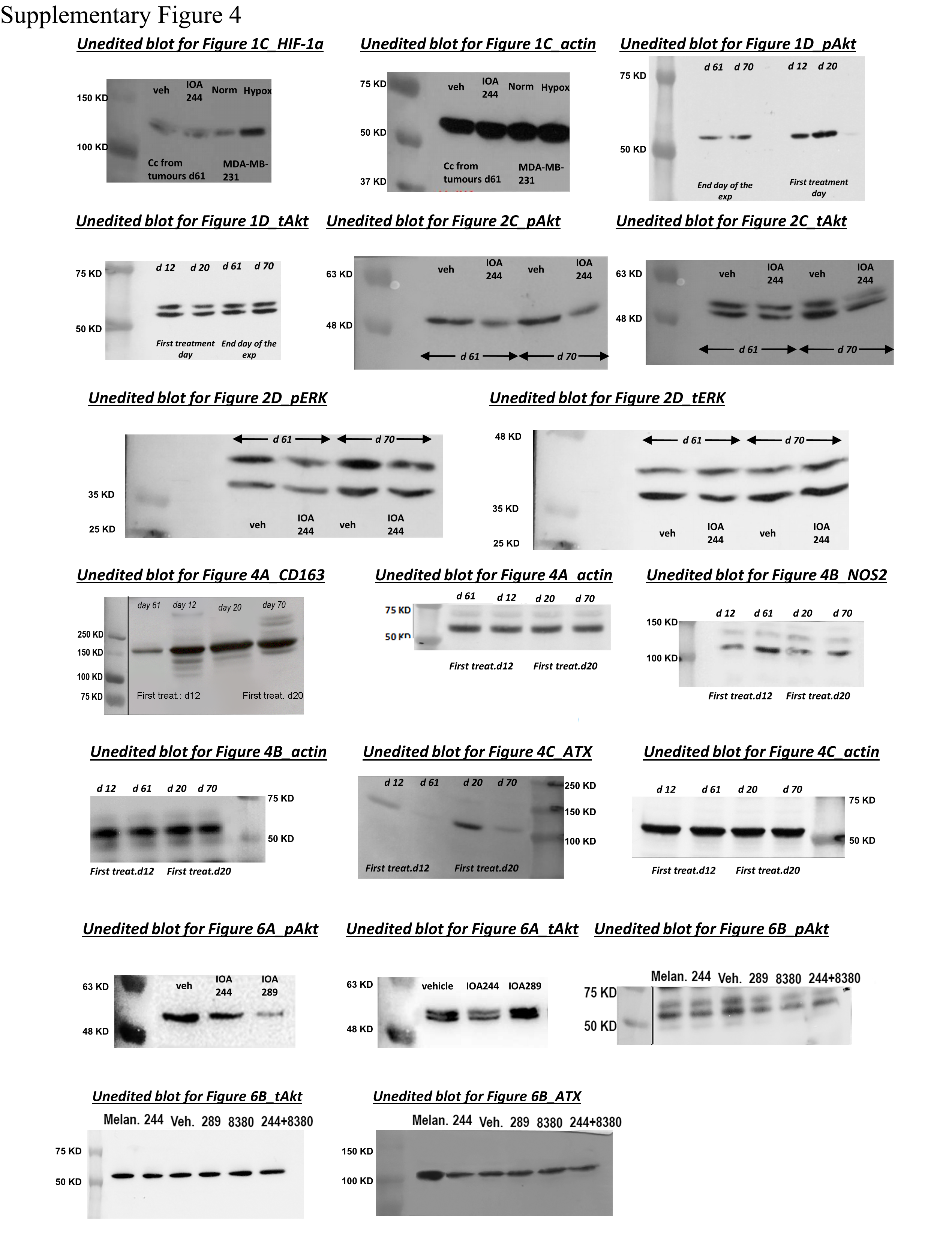

Supplement: Supplementary file 5 — Supp Fig4 [file 41420_2026_3073_MOESM5_ESM.tif]
